# A Rapid Response Thin-Film Plasmonic-Thermoelectric Light Detector

**DOI:** 10.1038/srep37564

**Published:** 2016-11-22

**Authors:** Ying Pan, Giulia Tagliabue, Hadi Eghlidi, Christian Höller, Susanne Dröscher, Guo Hong, Dimos Poulikakos

**Affiliations:** 1 Laboratory of Thermodynamics in Emerging Technologies, Institute of Energy Technology, Department of Mechanical and Process Engineering, ETH Zürich, CH-8092 Zürich, Switzerland; 2 Thomas J. Watson Sr Laboratories of Applied Physics, California Institute of Technology, Pasadena 91125, CA, USA; 3 greenTEG AG, Technoparkstrasse 1, 8005 Zürich, Switzerland

## Abstract

Light detection and quantification is fundamental to the functioning of a broad palette of technologies. While expensive avalanche photodiodes and superconducting bolometers are examples of detectors achieving single-photon sensitivity and time resolutions down to the picosecond range, thermoelectric-based photodetectors are much more affordable alternatives that can be used to measure substantially higher levels of light power (few kW/cm^2^). However, in thermoelectric detectors, achieving broadband or wavelength-selective performance with high sensitivity and good temporal resolution requires careful design of the absorbing element. Here, combining the high absorptivity and low heat capacity of a nanoengineered plasmonic thin-film absorber with the robustness and linear response of a thermoelectric sensor, we present a hybrid detector for visible and near-infrared light achieving response times of the order of 100 milliseconds, almost four times shorter than the same thermoelectric device covered with a conventional absorber. Furthermore, we show an almost two times higher light-to-electricity efficiency upon replacing the conventional absorber with a plasmonic absorber. With these improvements, which are direct results of the efficiency and ultra-small thickness of the plasmonic absorber, this hybrid detector constitutes an ideal component for various medium-intensity light sensing applications requiring spectrally tailored absorption coatings with either broadband or narrowband characteristics.

The efficient detection and measurement of light, specifically monochromatic (i.e. lasers) or broadband (i.e. white lamps) light in the visible and near-infrared (NIR) range, is a major undertaking in the engineering community and related industry. Indeed, the emission and absorption of photons are phenomena accompanying many physical processes such as broadband black body radiation^1^,^2^ from hot, macroscopic objects and wavelength-specific emission from fluorescent molecular^3^,^4^,^5^ or solid state single-photon sources^6^,^7^. The problem of measuring weak electromagnetic radiation across the entire visible and infrared range has been largely solved by devices such as avalanche photodiodes^8^ and superconducting bolometers^9^,^10^. In contrast, the measurement of medium/high-intensity light, with good temporal resolution and optional tunability of the spectral response, remains a challenge.

Thermoelectrics^11^,^12^,^13^,^14^,^15^,^16^,^17^,^18^ constitutes a class of important engineering materials that can be developed into sensors by exploiting the ability to transduce between thermal and electrical signals. By combining a thermoelectric TE element with a light absorption coating, light detection can be performed. For white light detection, a broadband absorber would be desirable. On the other hand, the use of spectrally selective coatings could enable close to monochromatic light detection, for specialty application or spectrometry. In order to develop an efficient and fast light sensor based on a thermoelectric device it is thus necessary to engineer a spectrally tailorable, high-absorption, low heat-capacity, and low radiative heat-loss material to efficiently mediate the photon-to-heat conversion step^19^.

Plasmonic nanostructures are ideal candidates for such an energy conversion task^20^,^21^,^22^,^23^,^24^,^25^,^26^. They enable effective light absorption, with absorber thicknesses in the sub-wavelength regime, 1–2 orders of magnitude thinner than conventional broadband absorber coatings^27^,^28^,^29^,^30^,^31^. Due to their small thickness, plasmonic absorbers have inherently small heat capacities, thus combining a high light-to-heat conversion efficiency^23^ with a fast response. In addition, their surface emissivity can be spectrally tuned to suppress infrared thermal radiation to minimize heat loss^23^. Lastly, light absorption by plasmonic structures can be designed to be either broadband or wavelength-selective^32^ by controlling the geometry of the nanostructures and combining different metals and dielectrics^29^,^30^,^31^. Together, these properties make plasmonic absorbers promising candidates for use as fast photon-to-phonon converters, which in combination with thermoelectric devices^33^,^34^ can lead to fast and efficient light detectors across a wide range of light intensities.

Indeed, in the field of infrared thermal light detection a variety of photonic and plasmonic absorbers have been realized either to overcome the poor intrinsic absorption of materials in this wavelength regime or to achieve high spectral selectivity^35^. Also, new materials such as graphene^36^ and carbon nanotubes^37^ have been exploited as tunable, ultra-low thermal capacity absorbers. On the other hand, much less attention has been devoted to the realization of thermoelectric-based visible/near-infrared light detectors, the reported response times being of the order of several seconds for light intensities of approximately 10^−4^ kW/cm^2^ (solar irradiation)^22^,^25^,^38^. Similarly, commercially-available TE-based light sensors measuring visible/NIR radiation with intensities of the order of few kW/cm^2^ still exhibit response times of the order of 1 second or greater^39^,^40^ due to the large heat capacity of the overall system.

Here, we explore the combination of a commercial thermoelectric detector with an ultra-thin, low heat-capacity plasmonic absorber for the rapid measurement of medium-intensity visible/NIR light. Our hybrid device exhibits linear response to light intensites up to approximately 1 kW/cm^2^ and response times in the range of few tens of milliseconds. In particular, we show that, while coating the device with a conventional absorber substantially increases the response time, the use of a plasmonic absorber leaves the response time of the initial thermoelectric system practically unchanged (only marginally increased), while at the same time markedly improving the radiation power-to-voltage sensitivity. Since the limiting factor in the speed of the thermoelectric-plasmonic light detector is the macroscopic thermoelectric component, further miniaturization of the basic thermoelectric voltage-generating unit would allow the realization of even shorter response times. Additionally, we show how the optical properties of plasmonic structures can be tuned to realize light absorbers with progressively broader spectral response by adjusting the geometrical parameters and material composition of the plasmonic coating.

## Results

**Figure 1:**
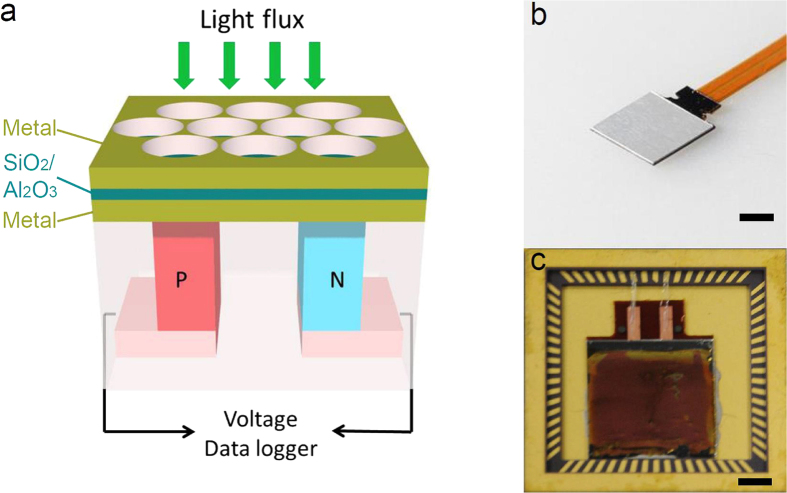
Thermoelectric-plasmonic hybrid light detector. (**a**) Schematic illustration of a thermoelectric sensor coated with plasmonic nanostructured light absorber. (**b**) Photograph of a sensor as-received from the manufacturer (greenTEG AG) and (**c**) photograph of the sensor coated with a broadband plasmonic absorber. The scale bars are 4 mm (**b**) and 2 mm (**c**), respectively.

The concept of the proposed hybrid plasmonic-thermoelectric light detection device is represented in Fig. 1, together with its actual implementation (Fig. 1a). On top of a commercial bismuth telluride thermoelectric device, gSKIN XP 26 9 C (provided by greenTEG AG, Fig. 1b), we fabricate an ultrathin plasmonic structure with tunable absorption properties and low thermal capacity (effectively a plasmonic absorber coating).

This absorber coating consists of a metal-insulator-metal (MIM) multilayer structure^23^,^29^,^30^,^31^. Silver and gold were used as the metals of choice for the fabrication of the absorbers. The thickness of the top, nanostructured layer and the bottom, uniform back reflector layer are both 100 nm. We use nanosphere lithography to pattern the top metallic layer into a hexagonal array of triangles or holes. The periodicity of the hexagonal crystal is 300 nm (holes center-to-center distance). The two metal layers are separated by a dielectric spacer consisting of either SiO_2_ or Al_2_O_3_. The entire multilayer structure is fabricated on top of a polished aluminum surface coupled to a commercial thermoelectric heat flux sensor (gSKIN XP 26 9 C). Details about the fabrication process are provided in Methods and Supplementary Information (SI). Figure 1b and c show two photographs of a thermoelectric sensor before and after coating with a broadband plasmonic absorber layer.

**Figure 2:**
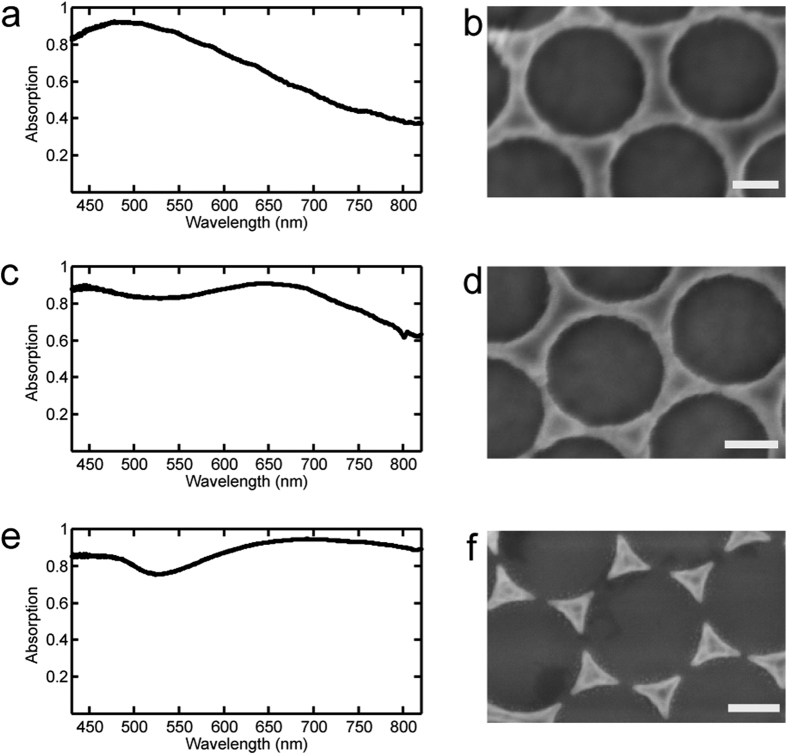
Measured absorption spectra for the narrow-band and broadband absorbers (left column), and scanning-electron microscopy (SEM) images on the right show unit cells of the fabricated structure for each case (scale bars represent 100 nm). Plasmonic absorber in (**a**) and (**b**) consists of the Ag/SiO_2_/Ag (100/34/100 nm), while absorber in (**c**) and (**d**) consists of Ag/Al_2_O_3_/Ag (100/34/100 nm). (**e**) and (**f**) show broader absorption spectrum with absorber consisting of Au/SiO_2_/Au (100/60/100 nm).

We demonstrate the tunability of the absorption spectrum of the plasmonic coating by fabricating structures with slight changes in the geometrical parameters as well as the spacer and metallic materials, while leaving unchanged the overall fabrication procedure. Three thermoelectric devices with the same metal/dielectric spacer/metal structure were fabricated. By changing the metallic material (either Au or Ag), dielectric spacer material (either SiO_2_ or Al_2_O_3_) and thickness and the degree of connectedness of the hexagonal patterns (Fig. 2), we achieved different absorption characteristics. The absorption measurements were performed using a home-built inverted microscope equipped with an air objective (numerical aperture, NA, 0.75) for exciting the sample and collecting the reflected light. Normal illumination was achieved by focusing the excitation light onto the center of the back-focal plane of the objective^41^. The reflected light was analyzed with a spectrometer (Princeton Instrument)^29^. The absorption spectrum, A, was calculated as A = (I − R)/I, where I and R are the incident illumination and reflected spectra, respectively.

The first absorber (sensor #1) is fabricated using Ag as metal, 34 nm of SiO_2_ as spacer layer and a hexagonal array of holes (Fig. 2a,b). The use of Ag and SiO_2_ allows the excitation of a plasmonic mode at short wavelengths. Indeed, this structure presents an absorption peak at 490 nm and its absorption drops below 75% for wavelengths longer than 610 nm and below 50% for wavelengths longer than 730 nm. The second absorber (sensor #2) is fabricated using Ag as metal, 34 nm of Al_2_O_3_ as spacer and a similar top pattern and exhibits a broader spectrum (red-shift of the plasmonic peak to 650 nm) with absorption dropping below 75% percent for wavelengths longer than 790 nm (Fig. 2c,d), due to the higher refractive index of Al_2_O_3_ compared to SiO_2_. Figure 2b and d show SEM images of these two triangle arrays have an average bottleneck width of 13–19 nm. The result of numerical simulations performed for these two sensors are discussed in section S3 of SI. The third absorber (sensor #3) is fabricated using Au as metal, 60 nm of SiO_2_ and an hexagonal array of disconnected triangles as front pattern^35^. It presents an ultra-broadband absorption spectrum with more than 75% absorption in the entire visible and near-infrared (NIR) range (from 400 nm up to 850 nm) (Fig. 2e,f).

A thermoelectric light sensor is characterized by two main figures of merit: sensitivity (voltage output per Watt input) and response time (characteristic time, τ). We thus tested our devices with respect to these parameters.

For the sensitivity measurements, we irradiated the sensor by modulating a green laser (CrystaLaser, λ = 532 nm) with a square wave with a period of 16 s, long enough to ensure that the voltage output reaches the steady state values within each half period (both on and off states). The laser power was varied from 32 mW to 110 mW and the irradiated area was approximately equal to ≈100 μm in diameter. The laser modulation was controlled using a shutter and the laser power was adjusted using neutral density filters. The voltage output from the sensor in response to the laser irradiation was recorded using a data logger (OM-DAQPRO-5300, as shown in Fig. 1a). More details can be found in the Methods and SI.

**Figure 3:**
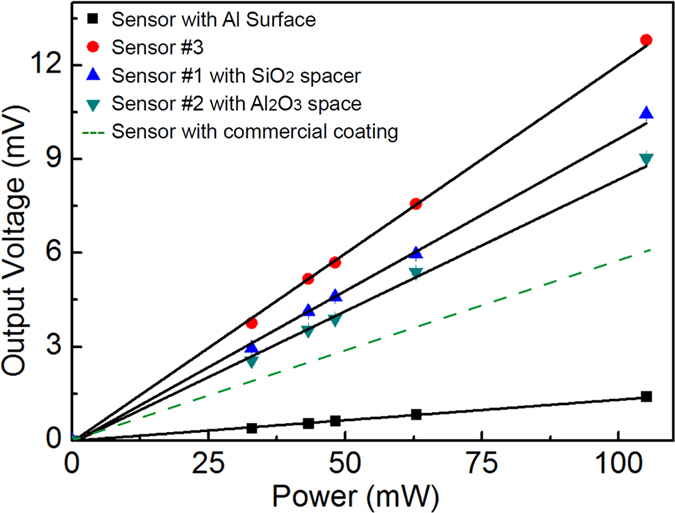
Voltage output of the light sensors as a function of the irradiation power.

Figure 3 shows representative voltage (mV)- radiation power (mW) characteristics of the light sensor without/with various plasmonic-mediated configurations. All devices exhibit linear responses as a function of the laser power. In the absence of any plasmonic coating, the sensitivity of the sensor with a polished aluminum surface is as low as 13.1 mV/W. In contrast, all plasmonic devices present significantly enhanced sensitivities. In particular, the sensors #1 and #2 (with Al_2_O_3_ and SiO_2_ spacers) present sensitivities of 82.8 mV/W and 94.8 mV/W, respectively, corresponding to a 6.3 and 7.2 fold enhancement. The broadband plasmonic detector presents the largest sensitivity of 116.1 mV/W, nearly 9 times larger than the uncoated detector. Different thermal resistivities of the individual plasmonic absorbers are most likely playing a secondary effect responsible for the variability in sensitivities achieved with different plasmonic coatings. Further optimization of the nanoscale heat generation and concentration with plasmonic nanostructures could thus open up additional possibilities for sensitivity enhancement and can be an interesting topic for future studies^42^. The DC noise level of the performed measurements is about 0.023 mV. This gives, according to Fig. 3, a noise-equivalent power of about 0.2 mW for the broadband sensor. For sensors without absorption layer, the noise is about 1.5 mW.

The commercial absorber that the company greenTEG AG uses for the sensors, is an inorganic ceramic coating applied with a spray process directly onto the sensor surface. Compared to the sensor with this commercial ceramic coating (gRAY B05-SC, olive green dashed line in Fig. 3, with sensitivity of 61.6 mV/W as specified by the company), sensor #3 with the broadband plasmonic absorber coatings exhibit a 2 fold sensitivity enhancement.

**Figure 4:**
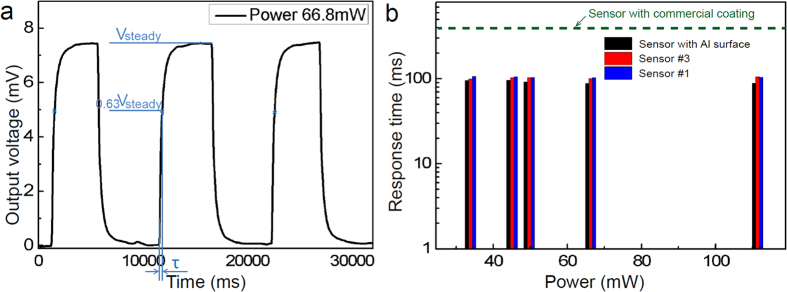
Characteristic thermal response of the broadband plasmonic absorber coated light flux sensor. (**a**) Time evolution of output voltage change in response to optical pulses with maximum incident optical power of 66.8 mW. (**b**) Characteristic time as a function of input power for different sensors at different incident power. The olive green dashed line is characteristic time of the sensor (gRAY B05-SC) with ceramic commercial coating specified by greenTEG AG.

We then characterized the transient response of the four devices by recording the output from the sensors under excitation with light pulses. We used a data acquisition rate of 2000 Hz, sufficient for capturing transient behaviors in the tens to hundreds of msec range. Figure 4a shows a typical time sequence, which we recorded at a given laser power (66.8 mW). From the time trace we can extract the two parameters of interests: the steady state voltage value, V_steady_, which we discussed in Fig. 3, and the transient response time, τ, of the device, defined as the time during which the output voltage reaches (1 − 1/e) times of its steady state value, V_steady_. For each case, we acquired at least 3–4 cycles of illumination in order to improve the accuracy of the time-constant (response time) determination.

Figure 4b shows that all the fabricated hybrid plasmonic-thermoelectric sensors display less than 10% increase in response time compared to the original sensor with polished aluminum surface (τ = 92±3 ms). Such marginally increased response time was verified at different laser illumination powers and is attributed to the small volume of the ultra-thin absorber coating and, therefore, its practically negligible heat capacity (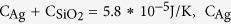

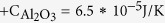
). In contrast, the same measurement performed for a sensor (gRAY B05-SC) with a commercial coating (20 μm) currently used by greenTEG AG for its detectors showed up to four times increase in response time (olive green dashed line in Fig. 4b) compared to the uncoated sensor. These results show that plasmonic structures are promising absorbers even for thinner and more optimized thermoelectric transducers with response times down to few milliseconds.

## Discussion

In conclusion, we demonstrated a visible-NIR, medium intensity, light flux sensor concept based on the combination of a thermoelectric device with an ultra-thin plasmonic absorber coating. The sensor coated with the plasmonic absorber achieved a sensitivity of 116 mV/W, nearly 9 times and 2 times higher than the sensitivity of the uncoated sensor and the sensor with commercial coating, respectively. Furthermore, while the commercial absorber coating substantially increases the response time of the sensor, application of the plasmonic sensor increased the response time by less than 10%. The absorption spectrum of the plasmonic coating and therefore, of the sensor, can be largely modulated by tuning the geometrical parameters of the absorber and the spacer material. Our work opens up a promising pathway towards achieving low-cost and fast light flux sensors for narrow-band or broadband light detection across the entire visible and near-infrared (NIR) wavelength range.

## Methods

### Sample Fabrication

The aluminum surfaces of the thermoelectric device (greenTEG, gSKIN XP 26 9 C) were lapped and then polished to reduce their surface roughness (see SI for details). A 100 nm-thick silver or gold back-reflector and a dielectric (SiO_2_ or Al_2_O_3_) layer were deposited successively on the polished aluminum surface of the sensors using electron-beam evaporation. The thermal deposition of the metal and dielectric film was conducted in a vacuum evaporator (Univex 500) at a pressure of 1 × 10^−7^ Pa with deposition rate of 0.1 nm/s. Successively, front hexagonal silver or gold pattern was fabricated using nano-sphere lithography and reactive ion etching. First, a closely packed monolayer of polystyrene beads (Duke Scientific, diameter 300 nm) is created using dip-coating: the sample is quickly immersed into a diluted bead solution (1 wt%, 10^−3^ M SDS) and then withdrawn with controlled velocity (≈2.5 μm s^−1^). The humidity is kept constant at ≈50%. Then, reactive ion etching is used to reduce the bead size (100 sccm Ar, 10 sccm O_2_, 50 W, 100 μbar). The bead size is controlled by adjusting the etching time. The final pattern is created by evaporating 100 nm of silver or gold through the bead mask and by successively removing the beads by low power ultrasonication (see SI for further details of the fabrication process).

### Optical Measurements

The absorption measurements were performed using a home-built inverted microscope equipped with an air objective (Zeiss 63x, numerical aperture NA 0.75) for exciting the sample and collecting the reflected light. Normal illumination was achieved by focusing the excitation light onto the center of the back-focal plane of the objective. The reflected light was analyzed with a spectrometer (Princeton Instrument). The absorption spectrum, A, was calculated as A = (I − R)/I, where I and R are the incident illumination and reflected spectra, respectively.

### Electrical Measurements

A green laser light (CrystaLaser, λ = 532 nm) was used for excitation. The output power was controlled by an intensity controller. The laser modulation was controlled using a shutter and the laser power was adjusted using neutral density filters (Thorlabs). The voltage output from the sensor in response to the laser irradiation was recorded using a data logger (OM-DAQPRO-5300). We used a data acquisition rate of 2000 Hz, sufficient for capturing transient behaviors in the tens to hundreds of milisecond range. From the voltage-time trace we extract the steady-state voltage value, V_steady_, and the transient response time, τ, of the device, defined as the time during which the output voltage reaches (1 − 1/e) times of its steady state value, V_steady_. For each case, we acquired at least 3–4 cycles of illumination in order to improve the accuracy of the time-constant (response time) determination. The DC detector noise was measured as the standard deviation of a time series of measured sensor output voltages in the absence of radiation excitation. For our detector, Johnson noise was found to dominate as the dynamic resistance of the system R_D = S^2*T/G = 0.96 Ohm, where S = (20 mV/K) is the Seebeck coefficient, T = 300 K is the operating temperature and G = 0.125 W/K is the thermal conductance, is much lower than the resistance between the leads of the device R > 55 Ohm. All measurements were performed in normal ambient conditions (air at atmospheric pressure and room temperature).

## Additional Information

**How to cite this article**: Pan, Y. *et al.* A Rapid Response Thin-Film Plasmonic-Thermoelectric Light Detector. *Sci. Rep.* **6**, 37564; doi: 10.1038/srep37564 (2016).

**Publisher’s note:** Springer Nature remains neutral with regard to jurisdictional claims in published maps and institutional affiliations.

## Supplementary Material

Supplementary Information
