# Supplementary material for: A Rapid Response Thin-Film Plasmonic-Thermoelectric Light Detector
Source: Sci Rep. 2016 Nov 22;6:37564. doi: 10.1038/srep37564 (PMC5118687; doi:10.1038/srep37564)
Supplement: Supplementary Information [file srep37564-s1.pdf]

Supporting information for:

"A Rapid Response Thin-Film Plasmonic-Thermoelectric Light Detector"

Ying Pan[1], Giulia Tagliabue[1,2a)], Hadi Eghlidi[1], Christian Höller[1], Susanne Dröscher[3], Guo Hong[1], Dimos Poulikakos[1a)]

[1]Laboratory of Thermodynamics in Emerging Technologies, Department of Mechanical and Process Engineering, ETH Zurich, Sonneggstrasse 3, 8092 Zürich, Switzerland

[2]Thomas J. Watson Sr Laboratories of Applied Physics, California Institute of Technology, Pasadena 91125, CA, USA

[3]greenTEG AG, Technoparkstrasse 1, 8005 Zürich, Switzerland

[a)]Authors to whom correspondence should be addressed. Electronic mail: giuliat@caltech.edu, dpoulikakos@ethz.ch

S1. Specifications of the thermoelectric device.

The dimensions of the thermoelectric device (gSKIN XP 26 9C, provided by greenTEG AG) are 10mm x 10 mm x 0.5 mm. The middle layer which contains the thermoelectric structure is 0.2 mm thick. Thermal resistance of the detector is 8 K/W. The Seebeck coefficient of the thermoelectric device is around 20-25 mV/K with ZT ca. 0.014.

The absorption spectra of the thermoelectric device with commercial coating (gRAY B05-SC) was shown in Fig. S1.

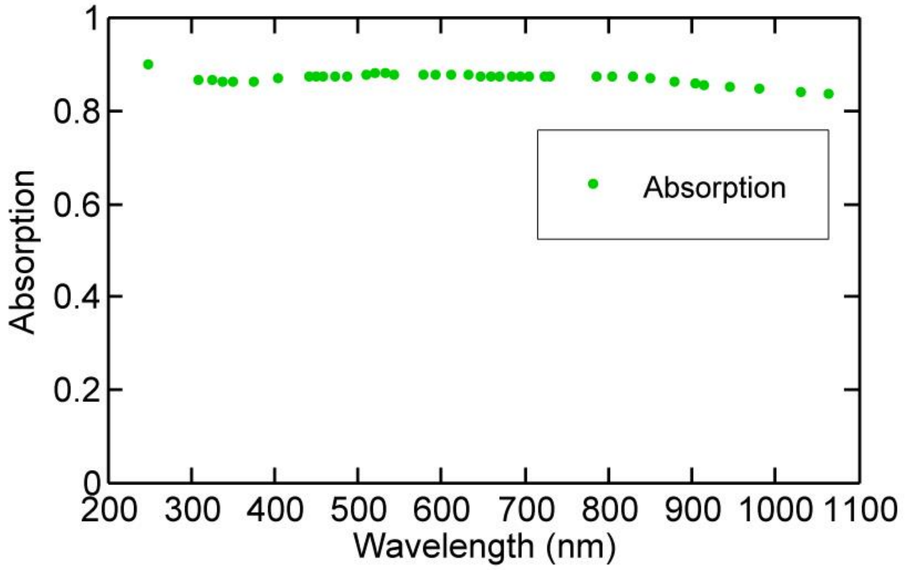

Figure S1: Absorption spectra of the gRAY B05-SC thermoelectric sensor change as wavelength.

S2. Sample Fabrication

Smooth aluminum surfaces were used as starting substrates in the fabrication. To achieve flat surfaces, the aluminum surfaces of the thermoelectric device (gSKIN XP 26 9C) were lapped and then polished to reduce their surface roughness. Below are the details about the lapping and polishing processes:

S2.1. Lapping steps:

The samples were first lapped for 18 min using solutions containing 9 µm Al_2O_3 particles with a lapping weight of 1024 g and stage rotation speed at 30 rpm to reduce sample thickness by 20 µm. Then, a solution containing smaller Al_2O_3 particles (3 µm) under a lighter lapping weigh of 400 g and with the same stage rotation speed of 30 rpm was used to gently reduce the extent of surface undulations on the aluminum surface over a period of 21 min.

S2.2. Polishing steps:

First, the aluminum surface was polished with a flow rate controllable lubricant liquid (1-2 drops/s) containing 3 μm diamond particles with polishing weight of 400 g and stage rotation speed at 60 rpm for 20 min.

Sequentially, the aluminum surface was further delicately polished with lubricant containing smaller diamond particles (1 μm) using otherwise identical conditions as the last step for 1 hour. By intermittently checking the surface, we could observe a gradual transition from an almost gray surface to a nearly mirror-like aluminum surface.

Finally, 0.02 μm colloidal silica (OPS) was used to finely polishing the aluminum surface at the same weight and stage rotation speed as the last step. Residual colloidal silica was then removed by *in situ* deionized water rinsing for a short time (30 s). At this point, the aluminum surface was completely mirror-like, with roughness *R*a (arithmetic average) measured to be 50±10 nm.

S2.3. Plasmonic nanostructure fabrication:

The absorber consists of a metal-insulator-metal (MIM) multilayer structure[1-4]. The MIM structure is composed of three layers as shown in Fig. S1. A 100 nm-thick silver or Au back-reflector and a 34 nm SiO_2 or Al_2O_3 layer 60nm SiO_2 layer were deposited succesively on the polished aluminum surface of the sensors using electron-beam evaporation. A front hexagonal silver or gold pattern was fabricated using nano-sphere lithography and reactive ion etching.

First, a closely packed monolayer of polystyrene (PS) beads (Duke Scientific) was created using dip-coating (Fig. S2a,b). The core of the dip-coating set-up is the linear micro stage (M-112.1 DG, PI-Physik Instrumente) controlled by a DC servo motor (C-863.11, PI-Physik Instrumente). These two components ensure a smooth movement even at very low speeds, necessary for preventing disturbances of the meniscus motion due to nonsmooth motion. The dip-coating set-up is placed inside a closed plexi-glass chamber, which is equipped with humidity and temperautre sensors.

Second, a reactive ion etching (RIE) process in an atmosphere of Ar and O_2 was used to reduce the bead diameters (Fig. S2c,d). Scanning electron microscopy (SEM) images in Fig. S2b and S2d show homogeneously self-assembled PS bead arrays covering large areas before and after RIE etching, respectively. The PS beads diameter in Fig. S2b and S2b is 300±10 nm and 290±10 nm, respectively. The error bars are a combination of the uncertainty due to the size determination and the standard deviation of multiple measurements (typically 5-6).

Third, silver or gold is deposited onto the sensor using electron beam evaporation (Fig. S2e,f). Finally after lift-off process, this forms a film with an array of connected or disconnected (depending on RIE etching time) triangles (Fig. S2g,h).

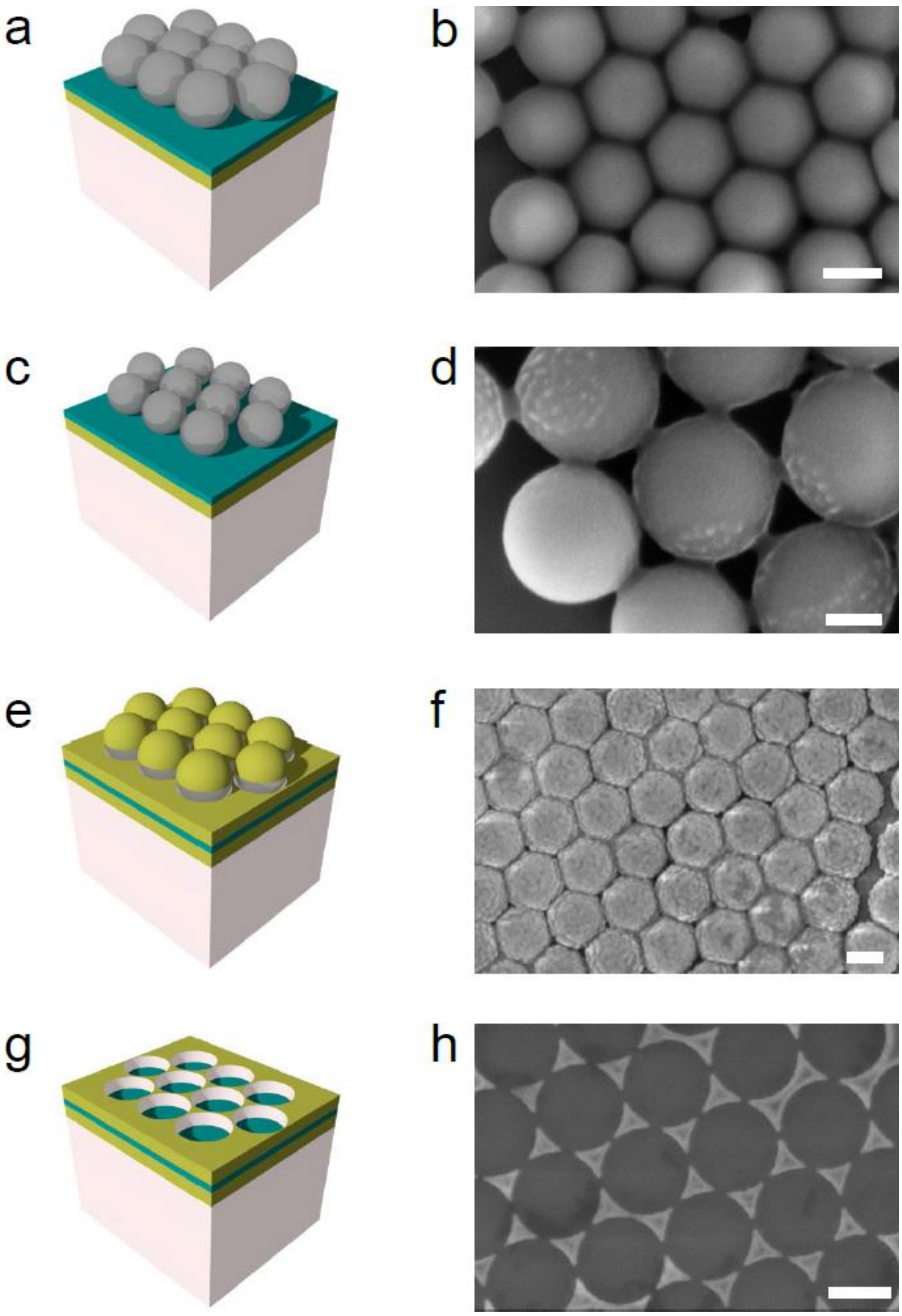

Figure S2: Ultrathin plasmonic absorber for thermoelectric device. (a) and (b) are closely packed monolayer of polystyrene beads. After a short reactive ion etching, the size of polystyrene beads was reduced as shown in (c) and (d). (e) and (f) are polystyrene beads covered by evaporated silver. (g) and (f) are silver triangle structures after the lift-off process. Scale bars in (b), (d), (f) and (h) show 200, 100, 200 and 200 nm, respectively.

S3. Simulation of plasmonic absorption

The geometry and materials of the broadband absorber have been discussed and optimized in our previous work (Ref. 35 in the main text). Figure S3 instead shows the calculated absorption spectra for the Ag/SiO_2/Ag (absorber #1) and Ag/Al_2O_3/Ag (absorber #2) (red curves). We see that, though leaving all the geometrical dimensions unaltered, the narrow-band peak absorption red-shifts from approx 500 nm to 600 nm due to the larger refractive index of Al_2O_3 (approx 1.75) compared to that of SiO_2 (approx 1.45). Experimentally we observe an analogous red-shift of the plasmonic mode. However, the spectral features of the experimentally measured absorption spectra are considerably broadened as compared to simulations. Two different reasons can be identified for this discrepancy. On the one hand, larger absorption is likely to originate from the increased imaginary part of the Ag permittivity (increased losses), due to small grain boundaries and surface roughness of the e-beam deposited Ag layers[5]. On the other hand, unavoidable fabrication imperfections are responsible of the spectral shift and further broadening of the plasmonic resonance peak. Indeed, as discussed in Ref. 35 and 37 in the main text, the periodicity of the structure (size of the polystarene beads) and the width of the Ag bridges in between the triangles are very important for controlling the position of the resonance. In the realized large area samples, there is a spread in both these two parameters due to the size dispersion of the polystyrene beads themselves and the non-homogeneities of the beads etching process. Figure S3.a,b shows how, starting from the idealized design geometry (red circle), accounting for these two effects allows to recover the progressively broadening spectrum of the experimental structures (blue diamond/orange square).

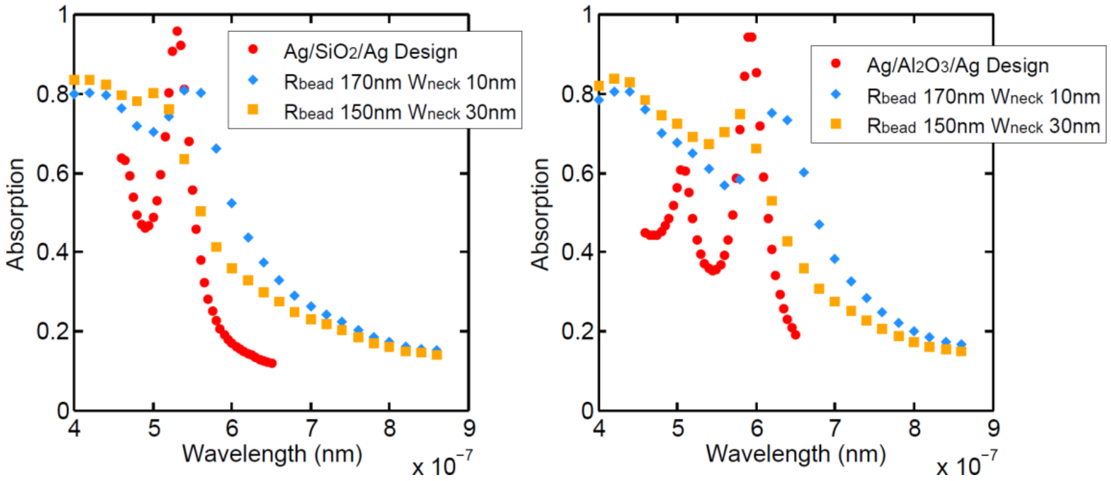

Figure S3. Absorption spectrum for various plasmonic absorbers. a) sensor #1 Ag/SiO_2/Ag and b) sensor #2 Ag/Al_2O_3/Ag. In both cases the red circle points show the idealized geometry calculation. Instead the blue diamond and orange square take into account increased lossed in Ag (epsilon_imaginary ≈ 3* epsilon_imaginary_Palik) as well as changes in the beads radius (150 nm and 170 nm) and neck widths (10 nm and 30 nm).

S4. Sensitivity and response time measurement

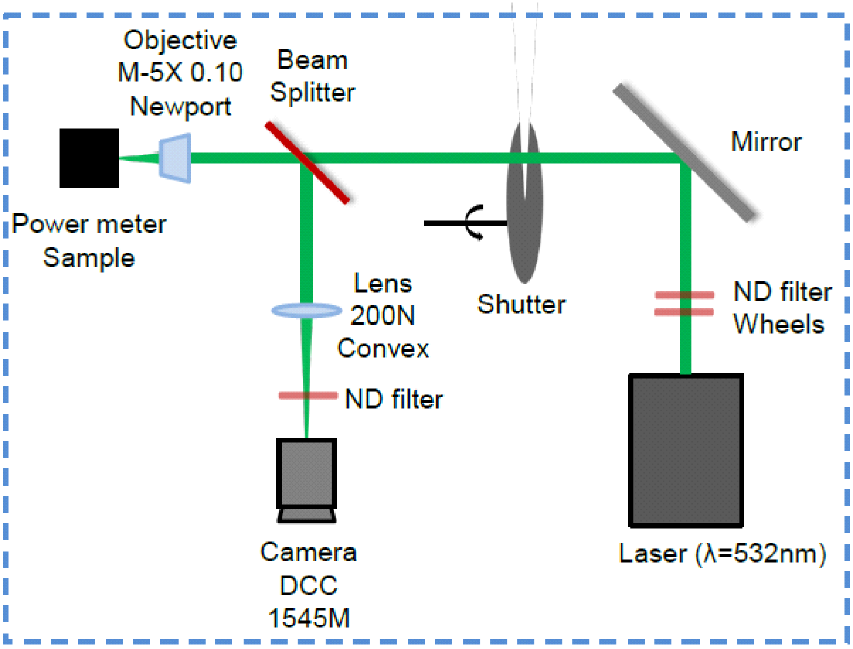

Figure S4. Optical and electrical measurement setup. The dashed square represents the optical shielding.

Experimental set-up used to characterize the sensitivities and response times (the two main performance parameters of a light detector) is illustrated in Fig. S4. A green laser light (λ= 536 nm) was used for excitation (Fig. S4, bottom-right). The output power was controlled by an intensity controller. Before each experiment, the laser was allowed to reach thermal equilibrium by outputting at 110 mW over 30 min. To achieve various lower output powers, optical neutral-density (ND) filters were employed (Fig. S3, above the Laser).

When testing output voltage from the hybrid light detector, the enclosure (shown as blue dotted line in Fig. S4) should be perfectly closed to avoid any fluctuations in power measurements and calibration. The sensor of the power meter appeared to be sensitive to temperature; therefore, for reproducible and accurate measurements, the setup was shielded and fully enclosed with black cardboard boxes.

S4.1 Sensitivity

The sensitivity of the light detector (in Fig. 3 of the main text), defined as the output voltage per mW of input light power, was calculated by averaging several equilibrium output voltages replicates at each incident light intensity. The resulting output voltages were plotted. A linear fit of the data yielded slope values corresponding to the sensitivities of the sensors.

S4.2 Response time

The response time τ is also an important property of a sensor (in Fig. 4 of the main text). The response time corresponds to the time that the sensor requires to react to the input power. The response time, τ, is usually given in units of milliseconds and is calculated as follows:

$$f(t = \tau) = \left(1 - \frac{1}{e}\right) f_\infty \approx 0.63 f_\infty \quad \text{(S1)}$$

where f_∞ is the steady state level of the signal after a sufficiently long period of illumination.

S4.3 Intensity capability of sensors

The irradiation power on the sample was measured by the power-meter after a beam splitter by considering the objective specification as shown in Fig. S3. The typical illumination intensity we tested is about 0.1 W. The illumination area on the sample had an area of 1*E-8 m^2. The intensity (W/m^2) of the light detector was obtained by taking the ratio of the power and illumination area. This results in an intensity of 1 kW/cm^2.

References

1. G. Tagliabue, H. Eghlidi, and D. Poulikakos. Rapid-response low infrared emission broadband ultrathin plasmonic light absorber. *Sci. Rep.*, 4, 7181 (2014).

2. G. Tagliabue, H. Eghlidi, D. Poulikakos. Facile multifunctional plasmonic sunlight harvesting with tapered triangle nanopatterning of thin films. *Nanoscale,* 5, 9957–9962 (2013).

3. G. Tagliabue, C. Höller, H. Eghlidi, D. Poulikakos. Proximal gap-plasmon nanoresonators in the limit of vanishing inter-cavity seperation. *Nanoscale*, 6, 10274–10280 (2014).

4. G. Tagliabue, D. Poulikakos, and H. Eghlidi. Three-dimensional concentration of light in deeply sub-wavelength, laterally tapered gap-plasmon nanocavities. *Appl. Phys. Lett.*, 108, 221108 (2016).

5. K. M. McPeak et al. . Plasmonic films can easily be better: rules and recipes. *ACS Photonics*, 2, 326-333 (2015).
